# Metabolic characterization of serum from mice challenged with *Orientia tsutsugamushi*–infected mites

**DOI:** 10.1016/j.nmni.2018.01.005

**Published:** 2018-02-22

**Authors:** C.-C. Chao, B.O. Ingram, W. Lurchachaiwong, W.-M. Ching

**Affiliations:** 1) Viral and Rickettsial Diseases Department, Infectious Diseases Directorate, Naval Medical Research Center, Silver Spring, MD, USA; 2) Uniformed Services University of the Health Sciences, Bethesda, MD, USA; 3) Metabolon Inc., Morrisville, NC, USA; 4) Entomology Department, Armed Forces Research Institute of Medical Sciences (AFRIMS), Bangkok, Thailand

**Keywords:** Chiggers, mass spectrometer, metabolites, metabolomics, mouse model, orientia tsutsugamushi, tryptophan

## Abstract

Scrub typhus is an acute zoonosis caused by the obligate intracellular Gram-negative bacterium *Orientia tsutsugamushi.* To better understand the host response elicited by natural infection by chigger feeding, ICR mice were infected by *Leptotrombidium chiangraiensis* (Lc1) chiggers, and the metabolic profiles of their serum were examined over several time points after initiation of feeding. ICR mice were infected by either naive Lc1 chiggers (i.e. not infected by *O. tsutsugamushi,* NLc1) or *O. tsutsugamushi*–infected Lc1 chiggers (OLc1). Serum was collected from both groups of mice at 6 hours and 10 days after initiation of feeding. Metabolites were extracted from the serum and analysed by ultra performance liquid chromatography–tandem mass spectrometry. The resulting ion/chromatographic features were matched to a library of chemical standards for identification and quantification. Biochemicals that differed significantly between the experimental groups were identified using Welch's two-sample *t* tests; p ≤ 0.05 was considered statistically significant. A number of biochemicals linked to immune function were found to be significantly altered between mice infected by the NLc1 and OLc1 chiggers, including itaconate, kynurenine and histamine. Several metabolites linked to energy production were also found to be altered in the animals. In addition lipid and carbohydrate metabolism, bile acid and phospholipid homeostasis, and nucleotide metabolism were also found to be different in these two groups of mice. Markers of stress and food intake were also significantly altered. Global untargeted metabolomic characterization revealed significant differences in the biochemical profiles of mice infected by the NLc1 versus OLc1 chiggers. These findings provide an important platform for further investigation of the host responses associated with chigger-borne *O. tsutsugamushi* infections.

## Introduction

*Orientia tsutsugamushi,* an obligate intracellular bacterium, is the aetiologic agent of scrub typhus, an acute febrile illness which can be transmitted by the bite of larvae of different species of trombiculid mites (e.g. chigger of *Leptotrombidium* mites). The mites can harbour the bacterium from the larval stage to adulthood and can maintain it via transovarial and transstadial transmission [1]. Rodents appear to be the natural hosts, while humans are accidental hosts. The disease is endemic to the Asia-Pacific region, where it accounts for up to 23% of all febrile illnesses [2]. In spite of its heavy disease burden in one of the most populated areas in the world, there is no vaccine available. The disease is characterized by fever, rash, eschar, pneumonitis and meningitis, and in some cases by disseminated intravascular coagulation that may lead to circulatory failure [3]. Although the disease can be effectively treated with doxycycline, timely and accurate diagnosis is often challenging because of its undifferentiated symptoms [4].

*Orientia* can infect a variety of mammalian cells *in vitro.* Human endothelial cells have long been thought to be the target of infection. This was confirmed by immunohistochemistry using autopsy tissues of suspicious cases of scrub typhus [5]. However, dendritic cells and monocytes rather than endothelium cells were shown to be the target cells in eschars of scrub typhus patients [6], suggesting the target cells at the initial biting sites of *Orientia* may be different from its target cells during subsequent dissemination and may influence its interaction with local host immune responses. Various mouse models have been developed to mimic the responses in patients. While intraperitoneal inoculation has been used to evaluate several vaccine candidates in mice [7], [8], [9], [10], additional routes for inoculation, including intradermal [11], [12], intravenous [13], [14], [15], [16] and footpad [17], have also been explored, along with their impacts on immunologic responses. Recently a mouse model using laboratory-reared, field isolated, *Orientia*-infected *Leptotrombidium chiangraiensis* (Lc1) chiggers as the inoculum was established, mimicking the natural route of infection [18]. Furthermore, these authors also established that infected chiggers can also cause infection in mice via intraperitoneal inoculation [19], [20]. A study utilizing this model to evaluate the leading vaccine candidate (a 56 kDa protein antigen) showed only a moderate protective effect [21]. Despite the advances in this mouse model with a natural vector (i.e. chiggers) to mimic the infection in rodents, not much is known about how the infection by *Orientia* affects the rodent and its metabolism during the early and late stages of infection.

In this study, ICR mice were infected by naive Lc1 (NLc1) chiggers and *Orientia*-infected Lc1 (OLc1) chiggers according to an established method [18]. The mice were monitored for up to 15 days after initiation of feeding (PIF). Serum samples were collected from both NLc1 and OLc1 chigger-infected mice, and the quantification of various metabolites was performed using ultra performance liquid chromatography–tandem mass spectrometry (UPLC-MS/MS). We observed significant alterations in several biochemicals linked to immune function and energy production. Additionally, there were also changes in metabolites reporting on food intake status, lipid and carbohydrate metabolism, and bile acid, phospholipid and nucleotide homeostasis. Our study is novel in that it is the first to describe the metabolic response mounted by laboratory animals in response to chigger-borne *O. tsutsugamushi* infections.

## Materials and methods

### Chigger feeding challenge in ICR mice

The animal protocol (PN #12-12), ‘Maintenance of the *Leptotrombidium* Larval Mite Colonies: Chigger Feeding on ICR Mice (*Mus musculus*),’ was approved by the AFRIMS Institutional Animal Care and Use Committee. The procedures described by Lurchachaiwong et al. [18] were followed. Individual mice were anesthetized by injection of a mixture of ketamine, atropine and xylazine (final concentrations of 40 mg/mL ketamine, 2 mg/mL xylazine and 0.06 mg/mL atropine, dosed at 0.1 to 0.2 mL/100 g mouse body weight). One Lc1 chigger was placed into the inner ear of one anesthetized female mouse. During the initial chigger feeding, mice were placed in special holding cages (11 cm long, 5 cm wide and 7 cm high) designed to restrict mouse movement and reduce the chance of removing chiggers by grooming or scratching. Each cage was positioned above a pan of water to catch any chiggers falling off the mice. Three days later, all mice (five mice per group) were transferred to regular caging condition. One set of mice was infected by the NLc1 chiggers. Another two sets of mice were infected by the OLc1 chiggers. After initiation of chigger feeding, one set each of the NLc1- and OLc1-infected mice were humanely killed by CO_2_ at 6 hours and 10 days PIF. The other OLc1-infected mice group was monitored for up to 28 days PIF or until they were deemed nonresponsive (Table 1). Blood was collected by cardiac puncture, and tissue samples of lung, liver, spleen, kidney and brain were also collected.

**Table 1:** Experimental design for metabolomic profiling of ICR mice infected by *Leptotrombidium chiangraiensis* chiggers

Infection	No. of mice	Time after feeding to sample collection
NLc1	5	6 hours
	5	10 days
OLc1	5	6 hours
	5	10 days
	5	15 days^a^

Lc1, *Leptotrombidium chiangraiensis;* NLc1, naive Lc1 chiggers; OLc1, *Orientia tsutsugamushi*–infected Lc1 chiggers.

a Group of mice infected by OLc1 chiggers was monitored only for morbidity and mortality. All mice were deemed nonresponsive and were humanely killed by 15 days after initiation of feeding.

### Metabolomic analysis

The nontargeted metabolomic analysis was performed at Metabolon Inc. (Morrisville, NC, USA). Detailed descriptions of the platform, including sample processing, instrument configuration, data acquisition and metabolite identification, and quantitation, have been published previously [22], [23], [24], [25], [26]. In brief, the samples were extracted with methanol and the supernatants were analysed using four independent UPLC-MS/MS methods: (1) reverse-phase (RP) UPLC-MS/MS with positive ion mode electrospray ionization (ESI), optimized for more hydrophilic compounds, (2) RP/UPLC-MS/MS method with positive ion mode ESI, optimized for more hydrophobic compounds, (3) RP/UPLC-MS/MS method with negative ion mode ESI and (4) hydrophilic interaction liquid chromatography/UPLC-MS/MS with negative ion mode ESI. All methods used a Waters ACQUITY UPLC (Waters, Milford, MA, USA) and a Q-Exactive high-resolution/accurate mass spectrometer (Thermo Fisher Scientific, Waltham, MA, USA) interfaced with a heated ESI-II source and Orbitrap mass analyser operated at 35 000 mass resolution. The methods alternated between full scan mass spectrometry (MS) and data-dependent MS^n^ scans using dynamic exclusion. The scan range varied slightly between methods but generally covered 70 to 1000 *m/z*. The structures of the metabolites were identified by automated comparison of the ion features in the experimental samples to a reference library of chemical standard entries that included retention time, molecular weight (*m/z*), preferred adducts and in-source fragments, as well as associated MS spectra, and was curated by visual inspection for quality control using software developed at Metabolon Inc. [24], [25], [26].

### Data analysis

Peaks were quantified using area-under-the-curve measurements. After median scaling and imputation of missing values, statistical analysis of log-transformed data was performed by open-source R software (R Foundation for Statistical Computing, Vienna, Austria; http://www.r-project.org/). Biochemicals that differed significantly between the experimental groups were determined via Welch's two-sample *t* test; p ≤ 0.05 was considered statistically significant.

## Results

### General observations of chigger-infected mice

After placement of a single chigger on the individual mice, the chiggers stayed on the mouse up to 3 days PIF before becoming fully engorged and detaching. All mice infected by the OLc1 chiggers died within 15 days PIF, similar to previous observations [18]. Symptoms, including ruffled fur, appeared on day 9 PIF, and mice also became less active and consumed less food on day 9 PIF. IgG and IgM antibodies against the immunodominant 56 kDa protein antigen were detectable by enzyme-linked immunosorbent assay only after 10 days PIF in all mice infected by the OLc1 chiggers. Tissue tropism was demonstrated by quantitative real-time PCR based on the 47 kDa gene [27]. Detectable *Orientia* DNA was evident after 10 days PIF in mice infected by OLc1, with lung being the most affected organ (data not shown).

### Summary of altered biochemicals and metabolites

We identified a total of 834 biochemicals in the serum of chigger-infected mice. The numbers of biochemicals found to be significantly different (p ≤ 0.05) between the OLc1- and NLc1-infected groups as well as those approaching significance (0.05 < p < 0.10) at 6 hours and 10 days PIF is shown in Table 2. Notably, the number of significant differences increased sharply over the course of the infection, increasing from 40 to 374 between 6 hours and 10 days PIF.

**Table 2:** Number of biochemicals altered in OLc1 mice relative to NLc1 mice

Time after feeding	p ≤ 0.05		0.05 < p < 0.10	
	Total biochemicals	Increases/decreases	Total biochemicals	Increases/decreases
6 hours	40	27/13	44	27/17
10 days	374	108/266	78	34/44

Welch's two-sample *t* test was used to determine statistical significance of fold changes of each biochemical in mice infected by OLc1 relative to NLc1 chiggers.

Lc1, *Leptotrombidium chiangraiensis;* NLc1, naive Lc1 chiggers; OLc1, *Orientia tsutsugamushi*–infected Lc1 chiggers.

**Fig. 1:**
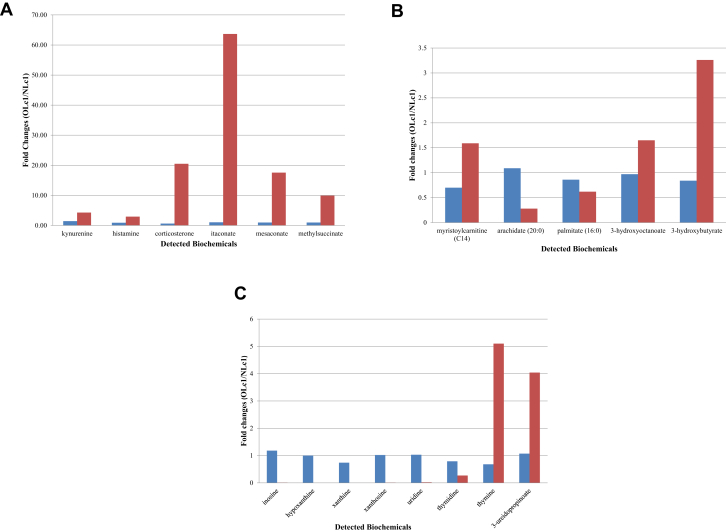
Fold changes of various metabolites at 6 hours (blue) and 10 days (red) PIF in mice infected by OLc1 chiggers relative to NLc1 chiggers. (A) Metabolites linked to infection. (B) Metabolites involved in fatty acid β-oxidation and ketogenesis pathways. (C) Metabolites involved in purine and pyrimidine metabolic pathways. Only fold changes observed at 10 days PIF is statistically significant (p ≤ 0.05, Welch's *t* test). Red bars for hypoxanthine and xanthine could not be seen clearly because of their extremely low fold changes of 0.003 and 0.0003, respectively. Lc1, *Leptotrombidium chiangraiensis;* NLc1, naive Lc1 chiggers; OLc1, *Orientia tsutsugamushi*–infected Lc1 chiggers; PIF, post initiation of feeding.

Metabolites linked to infection were up-regulated in mice with OLc1 chiggers. At 10 days PIF, the OLc1-infected mice exhibited significant elevations in several metabolites linked to immune function. These included kynurenine, histamine, corticosterone and itaconate, along with other structurally related C5 dicarboxylic acids, including mesaconate and methylsuccinate. As shown in Fig. 1(A), only negligible alterations were observed in these metabolites at 6 hours PIF. However, robust increases were observed for these metabolites at 10 days PIF, with the fold changes ranges ranging from 2.91 (histamine) to 64.1 (itaconate).

### Glucose and lipid utilization

At 10 days PIF, the mice infected by the OLc1 and NLc1 chiggers displayed significant differences in energy production pathways. Several glycolytic/gluconeogenic intermediates including glucose, pyruvate, lactate and several tricarboxylic acid (TCA) cycle intermediates were altered (Table 3), supporting the notion that the infection perturbed energy production in the mice. Perhaps as a compensatory mechanism, the OLc1-infected mice appeared to exhibit a greater reliance on lipids to fuel energy metabolism at this time. This notion is supported by the increases observed in acylcarnitine species (e.g. myristoylcarnitine, (C14)), free fatty acids species (e.g. arachidate (20:0), palmitate (16:0)), 3-hydroxy-fatty acids (e.g. 3-hydroxyoctanoate) and ketone bodies (e.g. 3-hydroxybutyrate (BHBA)) at 10 days PIF (Fig. 1(B)).

**Table 3:** Changes in metabolites involved in glucose utilization and TCA cycle activity

Subpathway	Biochemical name	Time after feeding	
		6 hours	10 days
Glycolysis	Glucose	0.99	0.59*
Gluconeogenesis	Phosphoenolpyruvate (PEP)	0.81	1.51*
Pyruvate metabolism	Pyruvate	1.31	0.53*
	Lactate	1.12	0.58*
Pentose phosphate	Sedoheptulose-7-phosphate	1.78	2.58*
TCA cycle	Succinylcarnitine (C4-DC)	0.60	0.63*
	Fumarate	1.21	0.33*
	Malate	1.12	0.39*

More biochemicals were detected in pathways than those listed.

Lc1, *Leptotrombidium chiangraiensis;* NLc1, naive Lc1 chiggers; OLc1, *Orientia tsutsugamushi*–infected Lc1 chiggers; TCA, tricarboxylic acid.

*Statistically significant difference (Welch's *t* test, p < 0.05) in fold changes of OLc1/NLc1.

As highlighted in Table 4, several additional classes of lipids were also altered. The OLc1-infected mice displayed significant decreases for a number of primary and secondary bile acid species at 10 days PIF. Bile acids are produced in the liver and play a role in emulsifying dietary fats, eliminating cholesterol and aiding in the excretion of hepatic catabolites; their lower abundance in the serum of the infected animals could thus correlate with impairments in lipid handling and/or hepatic function. In parallel with these changes, the infected mice also displayed decreases in several monoacylglycerol and diacylglycerol species (Supplementary Table S1), which is suggestive of a possible increase in triglyceride lipolysis rates. Lastly, the infected mice also displayed similar changes in many detected components of the glycerophospholipids, plasmalogens and sphingomyelins (Supplementary Table S2). Collectively, these changes are highly consistent with significant alterations in lipid synthesis and utilization near the onset of infection in the animals.

**Table 4:** Changes in primary and secondary bile acid metabolites

Subpathway	Biochemical name	Time after feeding	
		6 hours	10 days
Primary bile acid metabolism	Cholate	2.99	0.02*
	Glycocholate	1.51	0.11*
	Chenodeoxycholate	0.78	0.19*
	β-Muricholate	1.77	0.27*
Secondary bile acid metabolism	Deoxycholate	1.56	0.42*
	Ursodeoxycholate	0.91	0.10*
	12-Dehydrocholate	0.79	0.05*
	Ursocholate	0.98	0.16*

Lc1, *Leptotrombidium chiangraiensis;* NLc1, naive Lc1 chiggers; OLc1, *Orientia tsutsugamushi*–infected Lc1 chiggers.

*Statistically significant difference (p ≤ 0.05) in fold change (OLc1/NLc1) at 10 days after initiation of feeding.

### Perturbation of nucleotide metabolism in infected mice

Several alterations were observed in the nucleotide profiles of the infected mice. As highlighted in Fig. 1(C), the most significant changes were centred on metabolites linked to the breakdown and recycling of purines (e.g. inosine, hypoxanthine, xanthine and xanthosine) and pyrimidines (e.g. uridine, thymidine, thymine and 3-ureidopropionate). These late-developing differences may be attributable to changes in RNA/DNA synthesis and to breakdown and/or changes in nucleotide demand, possibly to fuel energy metabolism.

## Discussion

The OLc1-infected mouse model has recently been established [18] and has been used to evaluate vaccine efficacy [21]. This model well mimics infection observed in the field. Not much is known about the effect of *Orientia* infection on host metabolic activities. The increase in kynurenine levels as described herein for this infection model is particularly interesting, as it has been shown that the production of kynurenine is also elevated in patient serum samples as a result of increased indoleamine 2,3-dioxygenase activity [28]. Additionally, the production of itaconate has been associated with activation of the innate immune system [29]. Specifically, it has been linked to immunoresponsive gene 1 (*Irg1*), which is highly expressed in mammalian macrophages during infection [30]. The expressed protein of this gene functions as an inhibitor for isocitrate lyase, which is a key enzyme of the glyoxylate cycle involved in a metabolic pathway of importance reported for many pathogens during infection [31], [32]. In response to infection, some bacteria can degrade the itaconate to promote both their survival and infectivity in the host [33]. Although the above metabolic responses have been linked primarily to itaconate, methylsuccinate and methylfumarate could accumulate for similar reasons as well. Notably, these metabolites are structurally similar to itaconate and can be degraded or utilized by bacteria in similar manners [33], [34].

In addition to the above markers of infection, the infected mice also displayed significant increases in corticosterone at 10 days PIF. This metabolite, importantly, can affect both energy metabolism and the inflammatory state of the host [35], [36]. In the context of infection, it can aid in dampening the immune response by increasing the expression of anti-inflammatory genes and by inhibiting the expression of proinflammatory genes, allowing the body in turn to guard against hyperinflammation [36].

In parallel with the above changes, the experimental findings herein also suggest that the *Orientia* infection altered the major energy producing pathways in the host. There was evidence, for instance, of altered carbon flow through both the glycolytic and TCA cycle pathways (Table 2). Several classes of lipids were also altered in a pattern that was consistent with the β-oxidation pathway being up-regulated. It should be noted that these metabolic signatures correlated well with the increase in corticosterone noted above. Like other glucocorticoids, it can stimulate gluconeogenesis in the liver and inhibit glucose uptake by muscle, which leads to increases in fat breakdown and utilization. This phenotype may be an adaptive response by the host in response to feeding changes during the infection, as food consumption generally decreased as the infection progressed (data not shown). Interestingly, *Orientia,* unlike other rickettsia, does not have a β-oxidation system for fatty acid energy production [37]. Thus, it seems that *Orientia* may be taking advantage of the host fatty acid oxidation pathway to generate energy for its growth [38]. Similarly, the change in the nucleotide metabolites may also suggest the need for alternative energy production pathway for the host to utilize when infection is more evident, with less food intake. Finally, itaconate production (Fig. 1(A)) may also have an impact on cellular energy in the host because it is derived from the TCA cycle intermediate *cis*-aconitate via decarboxylation [33]. Moreover, experimental evidence has suggested that it can also inhibit glycolytic reaction in the host [39].

The increase in ketone body production in the infected mice in this study is also notable, as similar findings have been reported previously in other *Orientia* infection models [40]. Jung et al. [40], for instance, observed similar increases in BHBA in a BALB/c mouse model upon infection of *O. tsutsugamushi* via intraperitoneal inoculation. In their work, nuclear magnetic resonance analysis was used to quantify different metabolites in different tissues and serum. BHBA was one of the metabolites that increased in the infected mice relative to the uninfected control mice at days 4 or 7 after infection. While we only observed an increase at 10 days PIF, these independent findings are consistent with increased ketogenesis being a common metabolic response over the course of *Orientia* infection. Furthermore, almost all metabolites observed by Jung et al. in serum were also observed in this study (Supplementary Table S3). These consistent results suggest that the changes associated with these metabolites are independent of the genetic background of the mouse used, the inoculation route and methods used to monitor the level of these metabolites. Furthermore, a slight delay in time point (i.e. 4 or 7 days for intraperitoneal challenge and 10 days PIF for chigger infection) may be due to the time required for *Orientia* to enter the host via Lc1 inoculation [21]. Additionally, we also observed similar changes in phosphotidylcholine and phosphotidylethanolamine between the mice infected by OLc1 and NLc1 (Supplementary Table S2), as observed by Jung et al. The changes in phosphotidylcholine and phosphotidylethanolamine are likely related to the breakdown of *O. tsutsugamushi*–enveloping membrane in these infected mice [40].

Additional changes involving bile acids as well as glycerolipid and phospholipid metabolism were also observed at 10 days PIF (Table 4, Supplementary Tables S1 and S2). While it is difficult to pinpoint the exact mechanism or mechanisms responsible for these observations, these changes, when taken in combination, are consistent with a possible decline in complex lipid synthesis rates in the infected mice at or near the onset of illness and may support the ketogenic phenotype discussed above.

To our knowledge, this is first comprehensive study investigating the effect of chigger-borne *Orientia* infections on the host serum metabolome. The novelty of this study lies on the mouse model being the one that best mimics the route of natural infection. We observed alterations in several infection-linked markers in OLc1 mice, particularly at 10 days PIF. In addition, OLc1 animals also displayed significant differences in lipid and carbohydrate metabolism, bile acid homeostasis and nucleotide utilization. A more comprehensive study of additional time points after feeding with additional tissue samples should be conducted in order to gain better understanding of the changing dynamics of these metabolites and how the infection affects organ function.

## Appendix A Supplementary data

mmc1
