# Supplementary material for: Metabolic characterization of serum from mice challenged with Orientia tsutsugamushi–infected mites
Source: New Microbes New Infect. 2018 Feb 22;23:70–6. doi: 10.1016/j.nmni.2018.01.005 (PMC5913361; doi:10.1016/j.nmni.2018.01.005)
Supplement: mmc1 [file mmc1.docx]

**Supplemental Table 1**. Changes in the relative abundance of monoacylglyercols and diacylglycerol *

|  |  | Time post feeding | |
| --- | --- | --- | --- |
| Sub Pathway | Biochemical name | 6h | 10d |
| Monoacylglycerol | 1-myristoylglycerol (14:0) | 1.52 | **0.34** |
|  | 1-palmitoylglycerol (16:0) | 1.11 | 0.39** |
|  | 1-palmitoleoylglycerol (16:1) | **2.81** | **0.09** |
|  | 1-oleoylglycerol (18:1) | 2.70** | **0.14** |
|  | 1-linoleoylglycerol (18:2) | 2.35** | **0.12** |
|  | 1-linolenoylglycerol (18:3) | 2.05** | **0.08** |
|  | 1-arachidonylglycerol (20:4) | 1.00 | **0.40** |
|  | 1-docosahexaenoylglycerol (22:6) | 1.38 | 0.46 |
|  | 2-palmitoylglycerol (16:0) | 1.00 | **0.19** |
|  | 2-palmitoleoylglycerol (16:1) | 1.00 | **0.06** |
|  | 2-oleoylglycerol (18:1) | 1.51 | **0.08** |
|  | 2-linoleoylglycerol (18:2) | 2.13 | **0.07** |
|  | 2-arachidonoylglycerol (20:4) | 1.00 | **0.14** |
| Diacylglycerol | diacylglycerol (16:1/18:2 [2], 16:0/18:3 [1]) | 1.78 | **0.10** |
|  | palmitoyl-oleoyl-glycerol (16:0/18:1) [2] | 1.05 | **0.15** |
|  | palmitoyl-linoleoyl-glycerol (16:0/18:2) [1] | **2.06** | **0.22** |
|  | palmitoyl-linoleoyl-glycerol (16:0/18:2) [2] | 1.90** | **0.12** |
|  | palmitoleoyl-linoleoyl-glycerol (16:1/18:2) [1] | 2.12 | 0.19** |
|  | oleoyl-oleoyl-glycerol (18:1/18:1) [2] | 1.35 | **0.18** |
|  | oleoyl-linoleoyl-glycerol (18:1/18:2) [1] | 2.03** | 0.34 |
|  | oleoyl-linoleoyl-glycerol (18:1/18:2) [2] | 1.71 | **0.18** |
|  | linoleoyl-linoleoyl-glycerol (18:2/18:2) [1] | 2.08 | 0.28 |
|  | linoleoyl-linolenoyl-glycerol (18:2/18:3) [2] | 0.90 | **0.06** |
|  | oleoyl-arachidonoyl-glycerol (18:1/20:4) [2] | 1.77 | **0.17** |
|  | linoleoyl-arachidonoyl-glycerol (18:2/20:4) [1] | 1.66 | 0.33 |
|  | linoleoyl-arachidonoyl-glycerol (18:2/20:4) [2] | 1.32 | 0.17 |
|  | linoleoyl-docosahexaenoyl-glycerol (18:2/22:6) [2] | 1.36 | **0.09** |

*. The numbers represent the value (fold changes) obtained using the level of a given metabolite in the group of mice infected by the OLc1 chiggers divided by the level in the group infected by the NLc1 chiggers. The significant difference was determined as described in Materials and Methods. The bold numbers represent biochemicals with significant alteration (*p* <0.05).

**. Numbers represent biochemicals with less significant difference (0.05 < *p* < 0.1).

**Supplemental Table 2**. Changes in the relative abundance of glycerophospholipid, plasmalogen, and sphingomyelin *

|  |  | Time post feeding | |
| --- | --- | --- | --- |
| Sub pathways | Biochemical name | 6h | 10d |
| Phosphatidylcholine (PC) | 1,2-dipalmitoyl-GPC (16:0/16:0) | 0.76** | **0.44** |
|  | 1-palmitoyl-2-palmitoleoyl-GPC (16:0/16:1)* | 1.22 | **0.66** |
|  | 1-palmitoyl-2-stearoyl-GPC (16:0/18:0) | 0.67 | **0.43** |
|  | 1-palmitoyl-2-oleoyl-GPC (16:0/18:1) | 1.06 | 1.12 |
|  | 1-palmitoyl-2-linoleoyl-GPC (16:0/18:2) | 1.00 | **0.83** |
|  | 1-palmitoyl-2-gamma-linolenoyl-GPC (16:0/18:3n6)* | 1.62 | **0.46** |
|  | 1-palmitoleoyl-2-linoleoyl-GPC (16:1/18:2)* | 1.22 | 0.65 |
|  | 1-palmitoyl-2-arachidonoyl-GPC (16:0/20:4n6) | 1.05 | **0.72** |
|  | 1,2-distearoyl-GPC (18:0/18:0) | 0.51 | **0.46** |
|  | 1-stearoyl-2-oleoyl-GPC (18:0/18:1) | 0.80 | 1.33** |
|  | 1-stearoyl-2-linoleoyl-GPC (18:0/18:2)* | 0.92 | 0.92 |
|  | 1,2-dioleoyl-GPC (18:1/18:1) | 0.92 | 0.93 |
|  | 1-oleoyl-2-linoleoyl-GPC (18:1/18:2)* | 1.00 | **0.63** |
|  | 1,2-dilinoleoyl-GPC (18:2/18:2) | 1.12 | **0.31** |
|  | 1-linoleoyl-2-linolenoyl-GPC (18:2/18:3)* | 1.02 | 0.45 |
|  | 1-stearoyl-2-arachidonoyl-GPC (18:0/20:4) | 0.84 | 0.84 |
|  | 1-linoleoyl-2-arachidonoyl-GPC (18:2/20:4n6)* | 1.09 | **0.25** |
| Phosphatidylethanolamine (PE) | 1,2-dipalmitoyl-GPE (16:0/16:0)* | 1.00 | **0.38** |
|  | 1-palmitoyl-2-oleoyl-GPE (16:0/18:1) | 0.71 | **0.42** |
|  | 1-palmitoyl-2-linoleoyl-GPE (16:0/18:2) | 2.33 | 0.71 |
|  | 1-palmitoyl-2-arachidonoyl-GPE (16:0/20:4)* | 1.95 | **0.47** |
|  | 1-stearoyl-2-oleoyl-GPE (18:0/18:1) | 1.36 | 0.47** |
|  | 1-stearoyl-2-linoleoyl-GPE (18:0/18:2)* | 3.03 | 0.74 |
|  | 1-oleoyl-2-linoleoyl-GPE (18:1/18:2)* | 2.44 | 0.49 |
|  | 1,2-dilinoleoyl-GPE (18:2/18:2)* | 1.00 | 0.43 |
|  | 1-stearoyl-2-arachidonoyl-GPE (18:0/20:4) | 1.86 | 0.72 |
|  | 1-oleoyl-2-arachidonoyl-GPE (18:1/20:4)* | 1.34 | **0.40** |
|  | 1-linoleoyl-2-arachidonoyl-GPE (18:2/20:4)* | 1.00 | **0.42** |
| Phosphatidylserine (PS) | 1-stearoyl-2-arachidonoyl-GPS (18:0/20:4) | 1.06 | **0.33** |
| Phosphatidylinositol (PI) | 1-palmitoyl-2-linoleoyl-GPI (16:0/18:2) | 1.37 | 0.39** |
|  | 1-palmitoyl-2-arachidonoyl-GPI (16:0/20:4)* | 1.32 | **0.40** |
|  | 1-stearoyl-2-linoleoyl-GPI (18:0/18:2) | 1.27 | 0.49 |
|  | 1-stearoyl-2-arachidonoyl-GPI (18:0/20:4) | 0.75** | **0.72** |
| Plasmalogen | 1-(1-enyl-palmitoyl)-2-oleoyl-GPE (P-16:0/18:1)* | 0.39** | **0.41** |
|  | 1-(1-enyl-palmitoyl)-2-linoleoyl-GPE (P-16:0/18:2)* | 1.02 | **0.41** |
|  | 1-(1-enyl-palmitoyl)-2-palmitoyl-GPC (P-16:0/16:0)* | 0.69 | **0.33** |
|  | 1-(1-enyl-palmitoyl)-2-arachidonoyl-GPE (P-16:0/20:4)* | 0.73 | **0.45** |
|  | 1-(1-enyl-palmitoyl)-2-oleoyl-GPC (P-16:0/18:1)* | 0.73 | **0.39** |
|  | 1-(1-enyl-stearoyl)-2-oleoyl-GPE (P-18:0/18:1) | **0.58** | 0.42** |
|  | 1-(1-enyl-stearoyl)-2-linoleoyl-GPE (P-18:0/18:2)* | 0.90 | **0.33** |
|  | 1-(1-enyl-palmitoyl)-2-arachidonoyl-GPC (P-16:0/20:4)* | 0.73 | **0.38** |
|  | 1-(1-enyl-palmitoyl)-2-linoleoyl-GPC (P-16:0/18:2)* | 1.06 | **0.37** |
|  | 1-(1-enyl-stearoyl)-2-arachidonoyl-GPE (P-18:0/20:4)* | 0.72 | **0.41** |
| Sphingomyelin | sphingomyelin (d18:1/14:0, d16:1/16:0)* | 0.75 | **0.52** |
|  | sphingomyelin (d17:1/16:0, d18:1/15:0, d16:1/17:0)* | 0.76 | **0.54** |
|  | sphingomyelin (d18:2/16:0, d18:1/16:1)* | 0.69 | **0.61** |
|  | sphingomyelin (d18:1/17:0, d17:1/18:0, d19:1/16:0) | 0.69 | 0.65** |
|  | sphingomyelin (d18:1/18:1, d18:2/18:0) | 0.66** | 0.85 |
|  | sphingomyelin (d18:1/20:0, d16:1/22:0)* | 0.68 | 0.85 |
|  | sphingomyelin (d18:1/20:1, d18:2/20:0)* | **0.55** | 0.84 |
|  | sphingomyelin (d18:1/21:0, d17:1/22:0, d16:1/23:0)* | 0.76 | **0.40** |
|  | sphingomyelin (d18:1/22:1, d18:2/22:0, d16:1/24:1)* | 0.66 | 0.63** |
|  | sphingomyelin (d18:2/23:0, d18:1/23:1, d17:1/24:1)* | 0.68 | **0.44** |
|  | sphingomyelin (d18:1/24:1, d18:2/24:0)* | 0.59 | 1.33** |
|  | sphingomyelin (d18:2/24:1, d18:1/24:2)* | 0.64 | 1.10 |
|  | sphingosine | 0.58 | 1.02 |
|  | sphingosine 1-phosphate | 1.04 | 0.96 |
|  | sphingomyelin (d18:2/23:1)* | 0.80 | 0.54 |
|  | sphingomyelin (d18:2/21:0, d16:2/23:0)* | 0.52 | **0.26** |
|  | sphingomyelin (d18:2/24:2)* | 0.76** | 0.76 |
|  | sphingomyelin (d18:1/22:2, d18:2/22:1, d16:1/24:2)* | **0.67** | 1.31 |
|  | sphingomyelin (d18:0/20:0, d16:0/22:0)* | 1.00 | 0.32** |
|  | sphingomyelin (d18:0/18:0, d19:0/17:0)* | 0.41 | 0.46 |
|  | sphingomyelin (d18:2/18:1)* | 0.52 | 0.95 |
|  | sphingomyelin (d18:1/19:0, d19:1/18:0)* | 0.58 | **0.37** |

*. The numbers represent the value (fold changes) obtained using the level of a given metabolite in the group of mice infected by the OLc1chiggers divided by the level in the group infected by the NLc1 chiggers. The significant difference was determined as described in Materials and Methods. The bold numbers represent biochemicals with significant alteration (*p* <0.05).

**. These biochemcials are less significantly different (0.05 < *p* < 0.1).

**Supplemental Table 3**. Alteration in the relative abundance of various amino acids, their derivatives and other biochemicals in serum from mice infected by the OLc1 vs NLc1 chiggers*

| Biochemical name | Time post feeding | | Results from Jung et al. (40) |
| --- | --- | --- | --- |
|  | 6h | 10d | Up (+) or down (-) |
| Isoleucine | 1.05 | **0.68** | - |
| Leucine | 1.04 | **0.73** | - |
| Valine | 1.12 | **0.59** | - |
| Alanine | 1.05 | 0.74^a^ | - |
| Glutamate | 0.81 | **0.75** | - |
| Glycine | 1.02 | **0.72** | - |
| Lactate | 1.12 | **0.58** | - |
| Creatine | 1.03 | **1.21** | NC^b^ |
| Glucose | 0.99 | **0.59** | - |
| Choline | 0.71 | **0.81** | - |
| Betaine | 1.08 | 0.86^a^ | - |
| Glycerol | 0.81 | **0.18** | - |
| Fumarate | 1.21 | **0.33** | NC |

*. The numbers represent the value (fold changes) obtained using the level of a given metabolite in the group of mice fed on by the OLc1 chiggers divided by the level in the group fed on by the NLc1 chiggers. The significant difference was determined as described in Materials and Methods. The bold numbers represent biochemicals with significant alteration (*p* <0.05).

^a.^ These biochemcials are less significantly different (0.05 < *p* < 0.1).
